# Sterilization-Induced Property Changes in FDM-Printed Carbon Fiber-Reinforced Polycarbonate for Medical Device Applications

**DOI:** 10.3390/jfb17040159

**Published:** 2026-03-24

**Authors:** Abel Remache, Wilson Pavon, Oscar Jara Vinueza, Josseline Chicaiza, Jorge Mauricio Fuentes, Homero Cadena

**Affiliations:** 1 Carrera de Ingeniería Mecánica, Facultad de Ingeniería y Ciencias Aplicadas, Universidad Central del Ecuador, Quito EC170125, Ecuador; jmfuentes@uce.edu.ec (J.M.F.); hdcadena@uce.edu.ec (H.C.); 2 Facultad de Ciencias de la Ingeniería e Industrias, Universidad UTE, Quito EC170129, Ecuador; wilson.pavon@ute.edu.ec; 3 Facultad de Arquitectura y Diseño, Universidad de Las Américas UDLA, Quito EC170125, Ecuador; oscar.jara@udla.edu.ec; 4 Carrera de Diseño Industrial, Facultad de Ingeniería y Ciencias Aplicadas, Universidad Central del Ecuador, Quito EC170125, Ecuador; josseline_chicaiza@hotmail.com

**Keywords:** fused deposition modeling (FDM), carbon fiber-reinforced polymer (CFRP), additive manufacturing, sterilization effects, thermal stability, viscoelastic properties

## Abstract

Fused deposition modeling (FDM) of carbon fiber-reinforced polycarbonate (PC-CF) is increasingly used in medical applications due to its excellent strength-to-weight ratio and adaptability for custom geometries. However, sterilization is a critical step that may compromise the structural integrity of polymer composites. This study investigates the effects of two low-temperature sterilization methods—ethylene oxide (EO) and hydrogen peroxide vapor (HP)—on the mechanical, thermal, and viscoelastic properties of FDM-printed PC-CF parts. Characterization included tensile, impact, and hardness tests; thermomechanical analysis (TMA); and dynamic mechanical analysis (DMA). EO sterilization resulted in approximately 20% reduced elongation at break and lower glass transition temperature, indicating a loss of ductility and thermal stability. HP-treated samples showed reduced stiffness (16% in Young modulus) but increased *Tg* and reduced thermal expansion, suggesting improved dimensional stability. DMA results confirmed distinct viscoelastic behavior between treatment types. These findings provide evidence for selecting appropriate sterilization protocols for FDM-manufactured PC-CF components used in functional medical devices.

## 1. Introduction

Additive manufacturing (AM), particularly fused deposition modeling (FDM), has emerged as a disruptive technology with transformative potential in various fields, including biomedical engineering. Its ability to fabricate customized components with complex geometries from a wide range of materials has redefined the design and production of medical devices, enabling personalized solutions that meet the anatomical and functional needs of individual patients [1,2].

A critical challenge in the implementation of 3D-printed medical components is sterilization, a mandatory step to eliminate pathogenic microorganisms and ensure safety in clinical environments [3]. However, sterilization procedures especially those involving chemical or thermal agents may significantly alter the thermal, mechanical, and morphological properties of polymer-based materials, potentially compromising their structural integrity and long-term performance [4,5].

Among the advanced materials suitable for 3D printing in healthcare, carbon fiber-reinforced polycarbonate (PC-CF) has gained attention due to its superior mechanical strength, stiffness, and dimensional stability. These properties make PC-CF an attractive candidate for functional medical components such as surgical guides, orthotic structures, and support fixtures [6,7]. When processed by FDM, PC-CF offers a favorable strength-to-weight ratio and excellent thermal behavior, although its anisotropic, layered microstructure may influence its response to external post-processing treatments, including sterilization.

Despite the growing interest in PC-CF for medical applications, there is a notable lack of systematic studies assessing the effects of widely used sterilization methods on its functional properties. In particular, the influence of low-temperature sterilization techniques such as ethylene oxide (EO) gas and hydrogen peroxide vapor (HP) remains poorly understood. These methods are commonly employed in hospital settings due to their compatibility with thermosensitive devices, but they may induce chemical, morphological, or structural changes in polymer composites [8,9,10]. The absence of comprehensive data on their effects limits the safe and regulated deployment of FDM-fabricated PC-CF parts in clinical scenarios.

While sterilization effects have been extensively investigated in commonly used 3D-printed polymers such as PLA, ABS, and unfilled polycarbonate, carbon fiber–reinforced thermoplastic composites fabricated via FDM remain comparatively underexplored. The incorporation of short carbon fibers modifies heat transfer pathways, interfacial bonding mechanisms, stress distribution, and viscoelastic behavior, potentially altering the material’s response to chemical sterilization agents. Therefore, degradation mechanisms reported for neat polymers cannot be directly extrapolated to fiber-reinforced systems. Furthermore, direct comparative analyses between EO and HP sterilization in FDM-printed PC-CF composites are scarce, leaving uncertainty regarding their relative impact on structural and thermomechanical integrity.

To address this knowledge gap, the present study investigates the effects of EO and HP sterilization on the mechanical, thermal, and morphological behavior of PC-CF specimens fabricated by FDM. It is hypothesized that sterilization may induce physicochemical changes capable of altering the performance of the material. Accordingly, a full characterization was conducted before and after sterilization, including tensile, impact, and hardness tests; thermomechanical analysis (TMA); and microstructural evaluation.

By providing empirical evidence on material behavior under clinically relevant sterilization protocols, this research contributes to informed decision-making in the qualification of 3D-printed polymer composites for regulated medical use. The findings support the advancement of additive manufacturing technologies in healthcare by promoting safety, durability, and performance of printed components [11].

This paper is organized to provide a comprehensive analysis of the effects of sterilization on the mechanical, thermal, and viscoelastic behavior of carbon fiber–reinforced polycarbonate (PC-CF) manufactured via FDM. Section 2 details the experimental methodology, describing the five-stage protocol implemented for material fabrication, sterilization, and subsequent testing. Section 3 presents the results of the mechanical characterization, including tensile, impact, and hardness measurements, supported by statistical analysis to evaluate differences among sterilization conditions. Section 4 discusses the implications of the findings, emphasizing the effects of hydrogen peroxide and ethylene oxide sterilization on polymer matrix integrity, interfacial bonding, and overall viscoelastic stability. Finally, Section 5 summarizes the main conclusions and proposes future research directions to enhance the thermal resistance and sterilization durability of FDM-produced PC-CF composites.

## 2. Methodology

**Figure 1:**
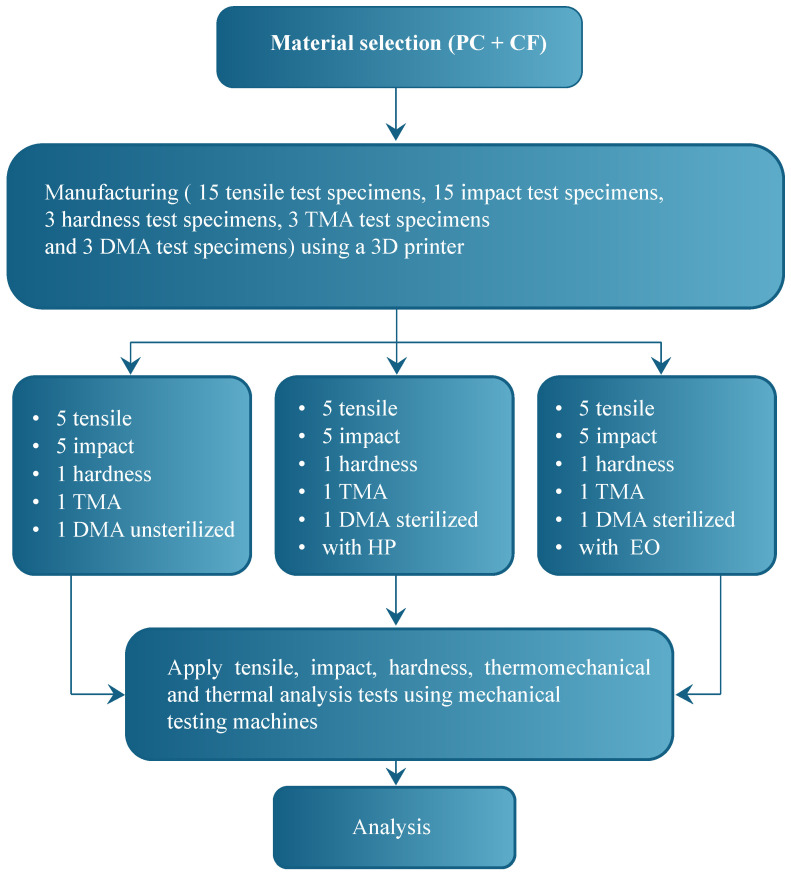
Flowchart of the methodological development used in the investigation.

This study followed a five-stage experimental protocol, as illustrated in Figure 1, to investigate the impact of sterilization on the properties of carbon fiber-reinforced polycarbonate (PC-CF) fabricated via FDM.

This study employed a five-stage experimental procedure, summarized in Algorithm 1, to evaluate the influence of ethylene oxide and hydrogen peroxide sterilization on the mechanical, thermal, and microstructural properties of FDM-printed carbon fiber–reinforced polycarbonate (PC-CF), following standardized fabrication, testing, and statistical analysis protocols.

**Algorithm 1** Fabrication and testing procedure for FDM-printed PC-CF under sterilization treatments.
1:Step 1: Material Selection2:Select Prusa Polymers PC-CF filament (1.75 mm diameter, ±0.04 mm tolerance) for its enhanced thermal resistance and dimensional stability compared to unmodified PC.3:Step 2: Sample Fabrication via FDM4:Model 36 specimens in SolidWorks 2024 according to ISO and ASTM geometries. Slice models using Smart3D software and print on a Smart3D Macro FDM printer at 275 °C nozzle, 110 °C bed, and 55 mm/s speed.5:Apply 80% infill for tensile, impact, and TMA/DMA samples, and 100% for hardness tests, with a rectilinear ±45° infill pattern.6:Step 3: Sterilization Treatments7:Divide samples into three groups: unsterilized (PC+CF), EO-sterilized (PC+CF-EO), and HP-sterilized (PC+CF-HP).8:Conduct EO sterilization in a SteriVac 5XL chamber at 55 °C for 60 min followed by 5 h 57 min aeration.9:Perform HP sterilization in a PlazMax P-110 system at 55 °C for 50 min followed by ventilation. Seal all samples hermetically after treatment.10:Step 4: Mechanical and Thermal Characterization11:Perform tensile testing (ISO 527-1) using Shimadzu AGSX, impact testing (ASTM D6110) using Ibertest-50, and Shore D hardness (ASTM D2240).12:Conduct TMA (ASTM E831) and DMA (TA ARG2) on representative specimens (*N* = 1 per group) to identify physicochemical trends and viscoelastic transitions, complementing the mechanical dataset.13:Step 5: Microstructural and Statistical Analysis14:Examine fracture surfaces under a stereomicroscope for morphological comparison across treatments.15:Perform statistical analysis of mechanical data using one-way ANOVA with Tukey’s post hoc test (95% confidence) in OriginPro 2024.


### 2.1. Material Selection

The experimental material was PC-CF filament supplied by Prusa Polymers A.S., Prague, Czech Republic, chosen for its improved heat resistance and dimensional stability compared to unmodified PC blends. According to the manufacturer, the filament has a net weight of 835 g, a total length of 303 m, and a diameter of 1.75 ± 0.04 mm [6].

### 2.2. Sample Fabrication via FDM

A total of 36 specimens were modeled in SolidWorks 2024 [12] following geometric specifications outlined in ISO 527-2 (Type 1BA) for tensile tests and relevant ASTM standards for impact and thermal characterization. STL files were sliced using Smart3D software Version 13.1 [13] and printed on a Smart3D Macro enclosed FDM, Delaware, USA, printer [14].

The printing parameters were strictly controlled to ensure reproducibility: a nozzle diameter of 0.4 mm, a layer height of 0.2 mm, and a flat XY printing orientation were utilized. The thermal settings included a nozzle temperature of 275 °C and a bed temperature of 110°C, with a print speed of 55 mm/s. Infill density was set at 80% for tensile, impact, and thermomechanical tests, and 100% for hardness measurements, applying a rectilinear infill pattern with alternating $\pm 45^{{}^{\circ}}$ angles to all samples.

**Figure 2:**
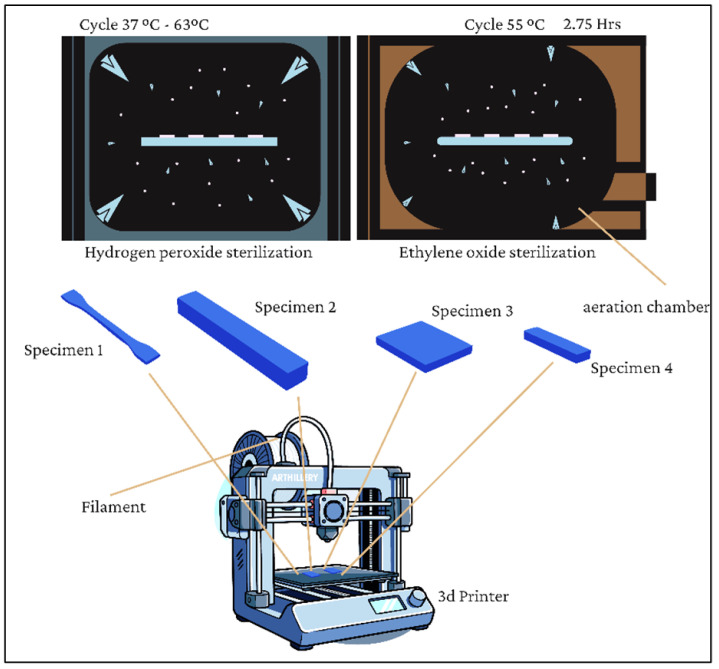
Diagram of the process used for specimen testing in this study.

Figure 2 Experimental workflow illustrating the fabrication of 3D-printed specimens and their subsequent exposure to hydrogen peroxide plasma and ethylene oxide sterilization prior to mechanical and thermo-mechanical characterization (hardness, tensile, impact, TMA and DMA).

### 2.3. Sterilization Treatments

**Figure 3:**
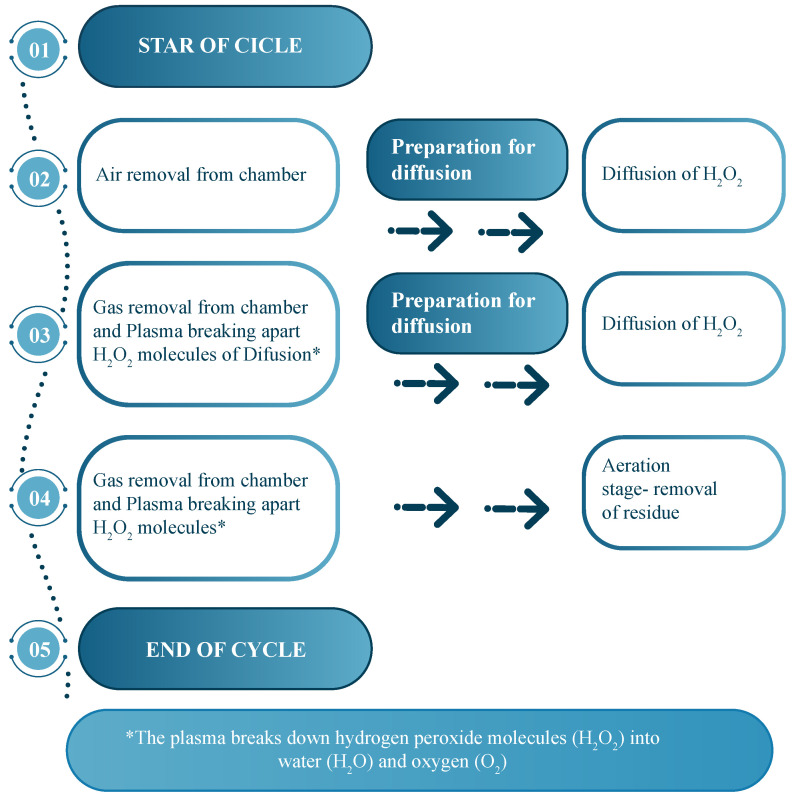
Hydrogen peroxide vapor sterilization cycle. The numbered labels (01–05) indicate the sequential stages of the process, from the start of the cycle to the final aeration stage.

Specimens were divided into three groups: unsterilized (control), sterilized with ethylene oxide (EO), and sterilized with HP, as shown in Table 1. The EO sterilization was performed using a SteriVac 5XL chamber [15] under a warm cycle at 55 °C and ambient temperature of 24.03 °C. Exposure lasted 60 min followed by aeration for 5 h and 57 min. Specimens were hermetically sealed post-treatment to avoid contamination. The HP sterilization was carried out in a PlazMax P-110 system from Tuttnauer (Israel) [16], described in Figure 3, using a low-temperature cycle at 55 °C with ambient conditions at 25 °C. Exposure time was 50 min, followed by a ventilation phase to extract residual agents. All treated specimens were hermetically sealed upon completion.

Following the sterilization processes, all specimens remained sealed in the original medical-grade packaging provided by the sterilization service provider to prevent external contamination or moisture absorption. The samples were stored in a climate-controlled environment at a temperature of 23±2 °C and a relative humidity of 50±5%, in accordance with ISO 291 standards [17]. All mechanical and thermal characterizations were performed within three business days post-sterilization to ensure that the evaluation reflected the immediate effects of the treatments while allowing for proper gas desorption.

### 2.4. Mechanical Characterization

#### 2.4.1. Tensile Testing

Tensile properties were assessed using a Shimadzu AGS-X Series universal testing machine (Shimadzu Corporation, Kyoto, Japan) [18], in accordance with ISO 527-1 [19], using a 50 kN load cell at a crosshead speed of 1 mm/min. The Trapezium X software, Version 2 [20] provided real-time stress–strain data for evaluating Young’s modulus, ultimate tensile strength, and elongation at break.

#### 2.4.2. Impact Testing

Charpy impact resistance was evaluated using an Ibertest-50 pendulum impact tester (S.A.E. Ibertest, Madrid, Spain) [21], following ASTM D6110-2024 [22]. The specimens were un-noticed. Dimensions were measured with a digital caliper, and absorbed energy values were used to calculate impact strength in kJ/m^2^.

#### 2.4.3. Hardness Testing

Hardness was measured using a Shore D durometer, following ASTM D2240 [23]. A constant pressure was applied, and the readings were taken after indentor stabilization.

#### 2.4.4. Thermal Characterization

Due to limited access to specialized thermal characterization equipment and logistical constraints, the TMA and DMA tests were conducted with a sample size of N=1 per group. Accordingly, these results should be interpreted as an exploratory assessment of thermal and viscoelastic behavior trends, providing qualitative support for the degradation mechanisms identified in the mechanical testing.

#### 2.4.5. Thermomechanical Analysis (TMA)

Thermal dimensional changes were evaluated via TMA, according to ASTM E831-19 [24], using a TA Instruments Q400 analyzer (TA Instruments, New Castle, DE, USA) [25]. Cubic samples (3 × 3 × 3 mm^3^) were subjected to a preload of 0.02 N and a compression load of 0.005 N. Heating was conducted from 25 °C to 200 °C at a rate of 5 °C/min to determine glass transition temperature (*Tg*) and linear Coefficient of Thermal Expansion (CTE).

#### 2.4.6. Dynamic Mechanical Analysis (DMA)

The viscoelastic properties were characterized using a TA Instruments AR-G2 rheometer (TA Instruments, New Castle, DE, USA) [26]. The instrument was configured in DMA mode and equipped with a specialized torsion/single-cantilever clamp for solid specimens. This setup enabled the precise measurement of the storage modulus ($G^{\prime }$), loss modulus ($G^{\prime \prime }$), and damping factor (tan(δ)) under a controlled temperature ramp from 25 °C to 200 °C at a frequency of 1 Hz. The use of this configuration is a validated method for assessing the thermomechanical transitions of 3D-printed polymer composites.

#### 2.4.7. Microstructural Analysis

Fractured surfaces of the Charpy-tested specimens were examined using a stereomicroscope to observe fracture patterns and morphology. Comparisons were made between unsterilized, EO-treated, and HP-treated samples to evaluate surface changes associated with each sterilization process.

#### 2.4.8. Statistical Analysis

Mechanical property data (tensile, impact, and hardness) were statistically analyzed using one-way ANOVA with Tukey’s post hoc test (p<0.05). Due to the limited sample size, TMA and DMA results were excluded from the ANOVA and were used for trend analysis and physical correlation with the mechanical findings.

## 3. Results Analysis

### 3.1. Mechanical Properties

The mechanical properties of the PC-CF specimens unsterilized, EO-sterilized, and HP-sterilized are summarized in Table 2. Each value represents the average of multiple replicates, with standard deviations and statistical significance indicated.

### 3.2. Tensile Strength

**Figure 4:**
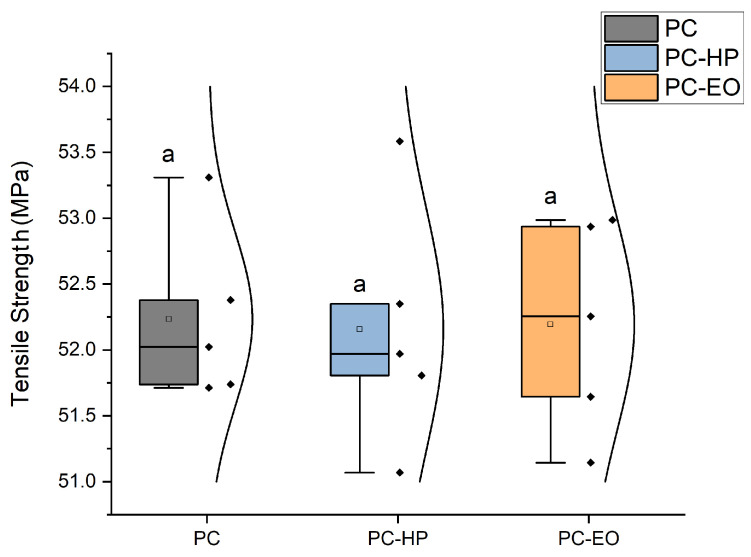
Ultimate tensile strength of PC-CF specimens under different sterilization conditions (unsterilized, EO-treated, and HP-treated). Square markers indicate mean values, while individual data points are overlaid for each group. Identical letters (a) denote that no statistically significant differences were found between groups (p<0.05), according to Tukey’s pairwise comparison.

As shown in Figure 4, there were no statistically significant differences (p>0.05) in ultimate tensile strength among the three groups. The average tensile strength was 53.0 ± 0.7 MPa for unsterilized PC-CF, 52.2 ± 0.4 MPa for HP-sterilized, and 52.2 ± 0.4 MPa for EO-sterilized samples. These results suggest that neither sterilization method caused significant degradation in tensile strength.

### 3.3. Young’s Modulus

**Figure 5:**
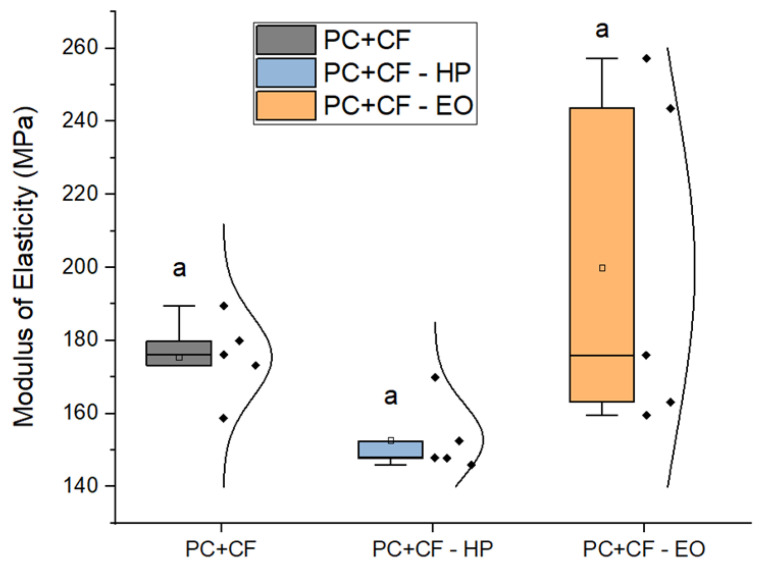
Young’s modulus of PC-CF specimens before and after sterilization. Square markers indicate mean values, while individual data points are overlaid for each group. Identical letters (a) denote that no statistically significant differences were found between groups (p<0.05), according to Tukey’s pairwise comparison.

Figure 5 illustrates the variation in Young’s modulus. Although no significant differences were detected via ANOVA, some dispersion was noted: the EO-sterilized group showed a slightly higher average modulus (199.9 ± 20.9 MPa), while the HP-sterilized group showed the lowest (152.8 ± 4.4 MPa). Unsterilized samples had an intermediate value of 175.5 ± 5.0 MPa.

### 3.4. Elongation at Break

**Figure 6:**
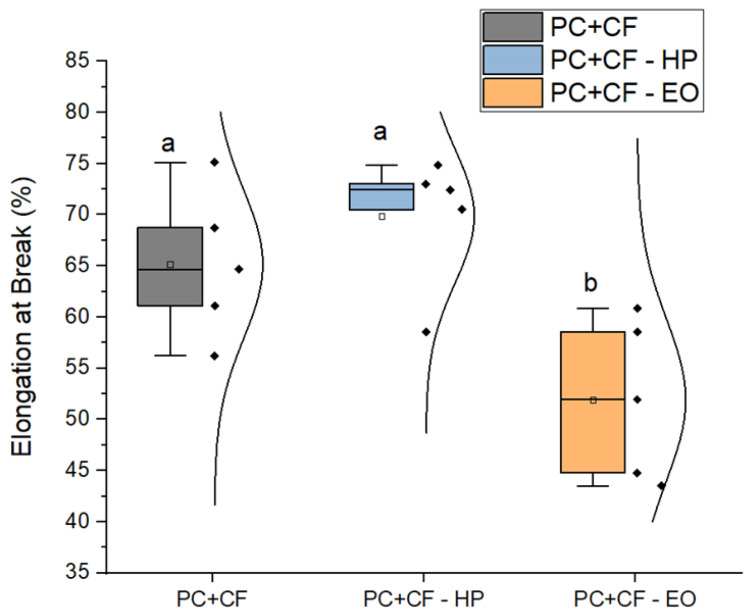
Elongation at break of PC-CF specimens under different sterilization conditions. Square markers represent mean values, and individual data points are overlaid for each group. Different letters indicate statistically significant differences between groups (p<0.05), according to Tukey’s pairwise comparison: groups sharing the same letter (a) are not significantly different, whereas groups labeled with different letters (b) show statistically significant differences.

The elongation at break (Figure 6) was significantly affected by EO sterilization. The EO-treated group exhibited a substantial reduction (51.9 ± 3.5%) compared to the unsterilized (65.2 ± 3.2%) and HP-treated (69.9 ± 2.9%) samples. Tukey’s test confirmed the statistical significance of this reduction (p<0.05), indicating a loss of ductility due to EO exposure.

### 3.5. Impact Resistance

**Figure 7:**
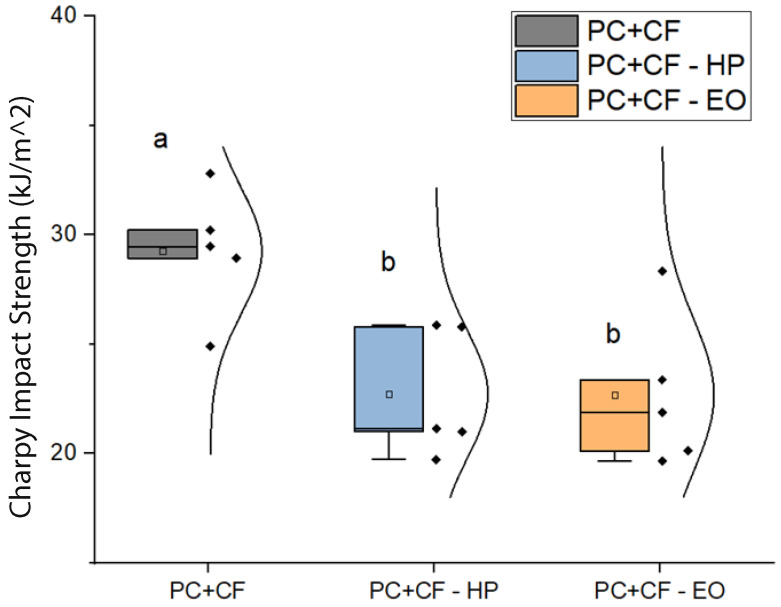
Charpy impact resistance of PC-CF specimens before and after sterilization. Square markers represent mean values, and individual data points are overlaid for each group. Different letters indicate statistically significant differences between groups (p<0.05), according to Tukey’s pairwise comparison: groups sharing the same letter (a) are not significantly different, whereas groups labeled with different letters (b) show statistically significant differences.

Figure 7 shows the Charpy impact resistance results. The unsterilized group exhibited the highest impact strength (29.3 ± 1.3 kJ/m^2^), whereas both sterilized groups (EO and HP) showed a statistically significant decrease (22.7 ± 1.3 kJ/m^2^). However, no significant difference was found between EO and HP groups, indicating that both sterilization methods reduce impact resistance to a similar extent.

### 3.6. Shore D Hardness

**Figure 8:**
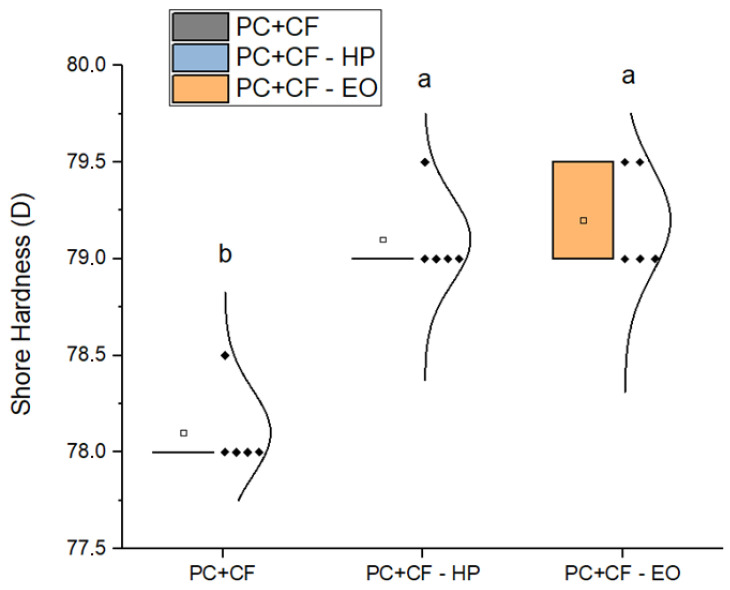
Shore D hardness for the three treatment conditions. Square markers represent mean values, and individual data points are overlaid for each group. Different letters indicate statistically significant differences between groups (p<0.05), according to Tukey’s pairwise comparison: groups sharing the same letter (a) are not significantly different, whereas groups labeled with different letters (b) show statistically significant differences.

As depicted in Figure 8, Shore D hardness increased slightly after sterilization. The values were 78.1 ± 0.1 for unsterilized samples, 79.1 ± 0.1 for HP-treated, and 79.2 ± 0.1 for EO-treated specimens. The increase was statistically significant (p<0.05), suggesting a potential surface densification or polymer matrix compaction effect.

### 3.7. Thermal Analysis

Although the sample size for the thermal analyses (N=1) limits the statistical generalization of the nominal values, the strong consistency observed between these profiles and the tensile and impact data (N=5) supports the validation of the physicochemical trends. For instance, the reduction in $T_{g}$ observed in the EO group is consistent with the loss of ductility and the microstructural changes reported in the previous sections, suggesting a robust physical correlation despite the absence of standard deviations for these specific tests.

### 3.8. Thermomechanical Analysis (TMA)

**Figure 9:**
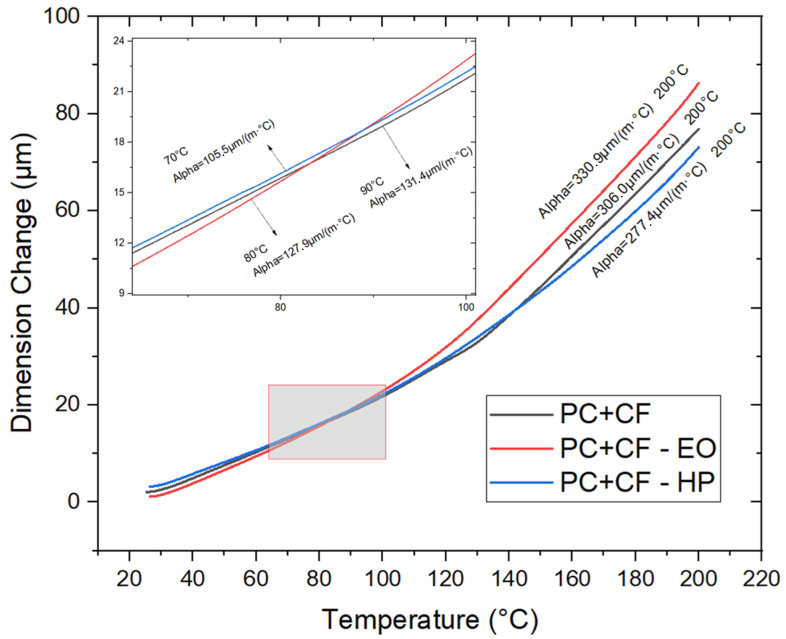
Thermomechanical analysis (TMA) curves of PC-CF specimens before and after sterilization, showing dimensional change versus temperature and highlighting variations in thermal expansion behavior and glass transition response among treatments.

The TMA results (Table 3 and Figure 9) show clear modifications in the thermal response of the PC-CF specimens after sterilization. EO exposure resulted in a noticeable decrease in the glass transition temperature ($T_{g}$), dropping from 136.6 °C to 126.9 °C. In contrast, HP treatment shifted the $T_{g}$ upward to 147.9 °C, suggesting enhanced thermal stability.

Changes in the CTE further highlight the different effects of both processes. The EO-treated samples exhibited higher thermal expansion both below and above $T_{g}$, reaching 330.9 µm/(m·°C) at 200 °C, which points to reduced dimensional stability at elevated temperatures. Conversely, the HP-treated specimens showed a lower CTE value (277.4 µm/(m·°C) at 200 °C), indicating a more stable thermomechanical response. Overall, these results suggest that while EO sterilization tends to compromise thermal performance, HP treatment may contribute to improved resistance under thermal loading conditions.

### 3.9. Viscoelastic Properties DMA

**Figure 10:**
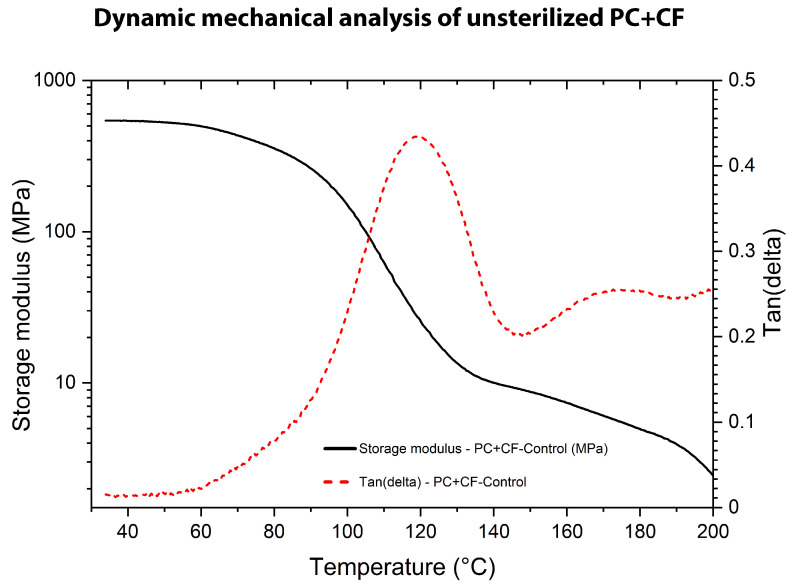
Dynamic Mechanical Analysis (DMA) of unsterilized PC-CF: storage modulus ($G^{\prime }$) and damping factor (tan(δ)) as a function of temperature.

**Figure 11:**
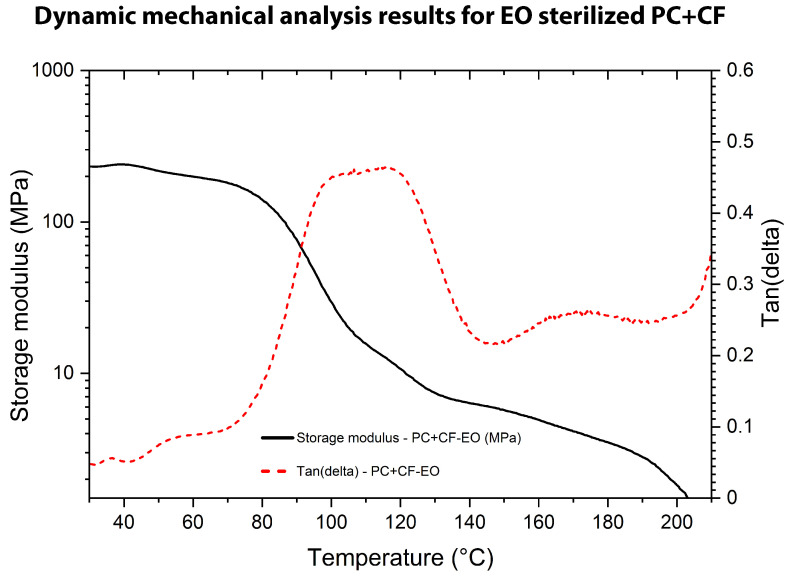
DMA results for EO-sterilized PC-CF: storage modulus ($G^{\prime }$) and tan(δ) as a function of temperature.

**Figure 12:**
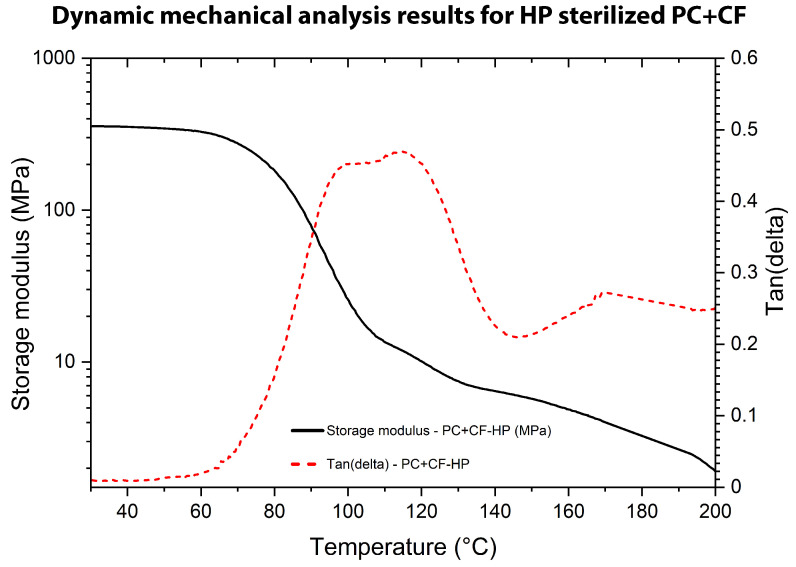
DMA results for HP-sterilized PC-CF: storage modulus ($G^{\prime }$) and tan(δ) as a function of temperature.

The DMA was carried out to assess the viscoelastic behavior of untreated and sterilized PC+CF samples, as shown in Figure 10, Figure 11 and Figure 12. Tests were performed in single-cantilever mode at 1 Hz with a heating rate of 3 °C/min from 25 °C to 250 °C. The storage modulus ($G^{\prime }$), loss modulus ($G^{\prime \prime }$), and damping factor (tan(δ)) were recorded as functions of temperature.

The DMA response of the unsterilized PC+CF sample (Figure 10) exhibits typical thermoplastic composite behavior. At low temperatures, high $G^{\prime }$ values (approximately 550 MPa) indicate a rigid glassy state in which the elastic component dominates over the viscous response ($G^{\prime }>G^{\prime \prime }$) As temperature increases, a pronounced decrease in $G^{\prime }$ occurs between approximately 90 °C and 120 °C, corresponding to the primary segmental relaxation of the polycarbonate matrix. The glass transition temperature determined by DMA ($T_{g,\mathrm{DMA}}$), defined as the temperature corresponding to the maximum of the tan(δ) curve, was 118.38 °C for the unsterilized material. Above this transition, a sharp reduction in structural rigidity was observed, consistent with the transformation from the glassy state to the amorphous rubbery region, where molecular mobility increases significantly.

The EO-treated sample (PC+CF-EO), in Figure 11, showed a marked reduction in its initial storage modulus, with $G^{\prime }$ values near 240 MPa in the glassy region, compared to approximately 540–550 MPa for the untreated specimen in Figure 10. This substantial decrease in stiffness indicates a weakened load-bearing capacity at low temperatures.

The viscoelastic transition also occurred at a lower temperature relative to the control sample, with the glass transition temperature determined by DMA ($T_{g,\mathrm{DMA}}$) measured at 114.32 °C. This shift toward lower temperature reflects increased molecular mobility and is consistent with partial plasticization or chain relaxation effects induced by ethylene oxide absorption. Furthermore, the smoother modulus decay and broader tan(δ) profile beyond $T_{g}$ suggest a diminished network recovery capacity and reduced intermolecular cohesion within the polymer matrix.

In contrast, the HP-treated PC+CF specimen (Figure 12) exhibits intermediate viscoelastic behavior, with an initial $G^{\prime }$ of approximately 360 MPa in the glassy region. This value is lower than that of the unsterilized material but higher than the EO-treated condition, indicating a moderate reduction in stiffness after hydrogen peroxide exposure. The glass transition temperature determined from the maximum of the tan(δ) curve was 114.73 °C, slightly lower than the unsterilized condition but comparable to the EO-treated sample. This modest reduction suggests that hydrogen peroxide sterilization induces moderate alterations in segmental dynamics without severely disrupting the viscoelastic transition of the polymer matrix.

The $T_{g}$ values obtained by DMA (118.38 °C for the control, 114.32 °C for EO, and 114.73 °C for HP) are lower than those measured by TMA. This difference is expected because DMA detects dynamic segmental relaxation under oscillatory loading conditions, which are highly sensitive to interfacial effects, microvoids, and anisotropic features inherent to FDM-printed composites [27]. In contrast, TMA determines $T_{g}$ from macroscopic dimensional expansion under quasi-static conditions. The difference between the $T_{g}$ values obtained by DMA and TMA may reflect the different sensitivities of these techniques to molecular mobility and dimensional changes. However, given that the thermal analyses were performed with a single specimen per condition, these observations should be interpreted as preliminary trends rather than definitive thermomechanical transitions.

Overall, the DMA results indicate that sterilization reduces the initial storage modulus and slightly shifts the glass transition toward lower temperatures, with EO producing the largest reduction in stiffness and HP showing a more moderate effect on viscoelastic stability.

### 3.10. Fourier Transform Infrared Spectroscopy of PC+CF

**Figure 13:**
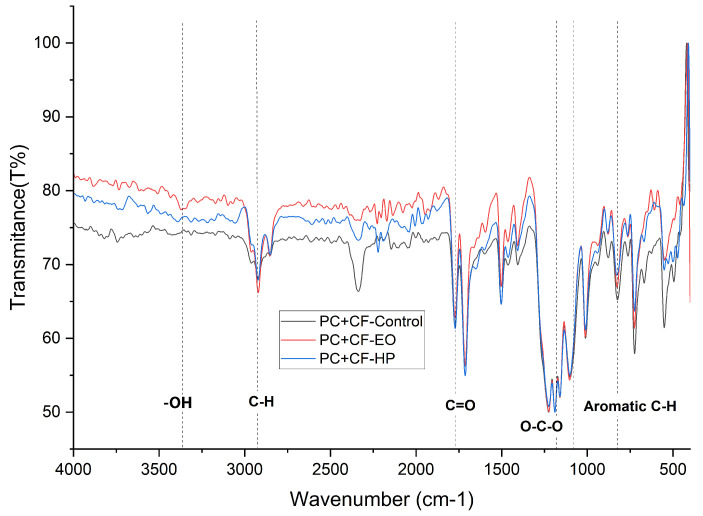
Fourier Transform Infrared Spectroscopy (FTIR) curves of 3D-printed (FDM) PC-CF sterilized with hydrogen peroxide (HP) and ethylene oxide (EO).

This section analyzes the chemical behavior of FDM-printed polycarbonate reinforced with carbon fiber (PC+CF) using Fourier Transform Infrared Spectroscopy (FTIR), with the objective of identifying the characteristic absorption bands of the polycarbonate matrix and detecting possible chemical modifications after sterilization with hydrogen peroxide (HP) and ethylene oxide (EO). The FTIR spectra obtained for the different treatment conditions are presented in Figure 13.

The interpretation of the vibrational bands was performed considering the typical wavenumber ranges reported for aromatic polycarbonates in reference tables of organic functional groups. Particular attention was given to the carbonate stretching vibrations, aromatic ring modes, and C–O–C linkages, which are sensitive to chain scission, oxidation, and plasticization phenomena that may occur during sterilization processes.

The FTIR spectrum of untreated PC+CF (Control) exhibits the characteristic features of bisphenol-A polycarbonate. A strong and sharp absorption band located at approximately 1770–1765 cm^−1^ corresponds to the stretching vibration of the carbonate carbonyl group (ν(C=O)), which serves as the primary structural fingerprint of the polymer backbone. Aromatic skeletal vibrations appear at around 1600 cm^−1^ and 1500 cm^−1^, confirming the presence of phenylene rings. The region between 2970 and 2870 cm^−1^ shows asymmetric and symmetric C–H stretching vibrations of methyl groups associated with the isopropylidene bridge. The asymmetric O–C–O stretching of the carbonate linkage is observed between 1220 and 1180 cm^−1^, while bands in the 1100–1000 cm^−1^ region are attributed to C–O stretching vibrations. Out-of-plane aromatic C–H bending modes appear near 830–750 cm^−1^. No additional significant bands attributable to carbon fiber oxidation are detected, indicating that the IR response is dominated by the polymer matrix.

After hydrogen peroxide sterilization, moderate spectral changes are observed. The intensity of the carbonate carbonyl band decreases slightly, suggesting partial oxidative attack or limited chain scission at the carbonate linkage. A subtle increase and broadening in the 3500–3200 cm^−1^ region indicate enhanced O–H stretching, consistent with the formation of hydroxyl end groups generated by oxidative degradation. Minor reductions in the methyl C–H stretching region (2970–2870 cm^−1^) further support oxidative processes affecting aliphatic segments. Additionally, small intensity variations in the fingerprint region (1220–1000 cm^−1^) suggest localized rearrangement or partial cleavage of the O–C–O backbone, likely restricted to surface layers.

In contrast, EO-sterilized PC+CF exhibits minimal spectral variation. The carbonate carbonyl band remains stable in both intensity and position, indicating preservation of the polymer backbone. Aromatic skeletal bands at 1600 and 1500 cm^−1^ show no significant changes, confirming the chemical stability of the aromatic structure. Only slight fluctuations in the fingerprint region are observed, likely associated with superficial interactions rather than structural degradation.

Overall, FTIR results indicate that HP induces low oxidative modifications in PC+CF, primarily affecting carbonate linkages and surface hydroxyl content, whereas EO sterilization did not produce detectable changes in the main FTIR absorption bands of polycarbonate, indicating that the primary chemical structure of the polymer backbone remained largely preserved. This apparent chemical stability does not exclude the possibility of physical modifications such as plasticization, residual EO absorption, or interfacial weakening within the layered FDM architecture, which may affect mechanical performance without generating detectable FTIR band shifts [28].

### 3.11. Microstructural Analysis

**Figure 14:**
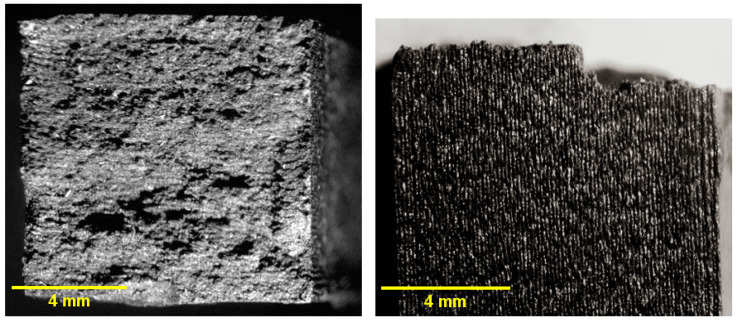
Fracture surfaces of unsterilized PC–CF under stereomicroscopy: (**left**) top view of the cross-section; (**right**) front view.

**Figure 15:**
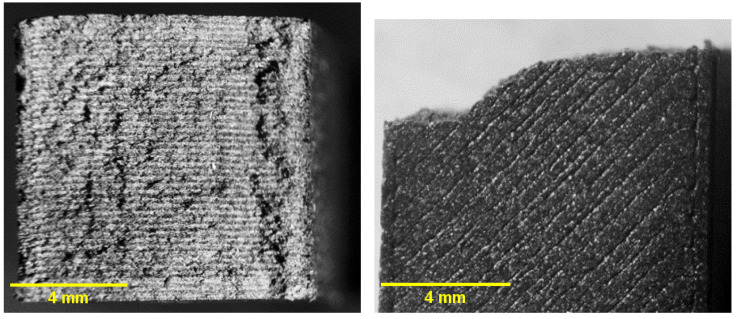
Fracture surfaces of HP-sterilized PC–CF: (**left**) top view of the cross-section; (**right**) front view.

**Figure 16:**
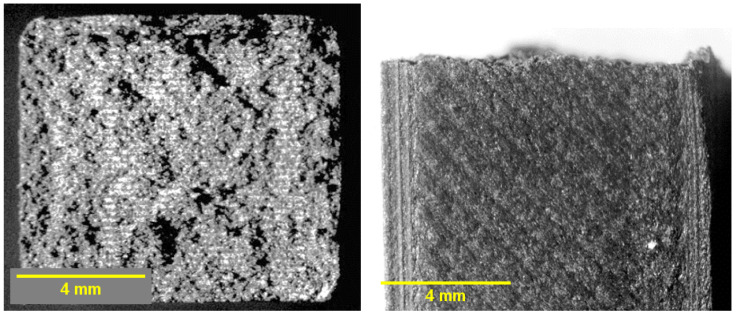
Fracture surfaces of EO-sterilized PC–CF: (**left**) top view of the cross-section; (**right**) front view.

Fracture surfaces of Charpy impact specimens were examined under stereomicroscopy following testing in accordance with ASTM D6110-2024. The specimens possess standardized cross-sectional dimensions of 10 × 10 mm, and the images presented in Figure 14, Figure 15 and Figure 16 correspond to full cross-sectional fracture views. All specimens exhibited predominantly brittle fracture patterns, though with distinct surface morphologies depending on the sterilization condition.

The unsterilized PC-CF specimens (Figure 14) exhibited a rough and irregular morphology consistent with brittle fracture and sudden crack propagation, with no evidence of sterilization-induced surface alterations. In contrast, the HP-treated samples (Figure 15) showed comparatively smoother facets and locally flattened regions, indicative of oxidative or thermal surface modification that may reduce interlayer cohesion, consistent with the measured decrease in impact strength and elastic modulus. The EO-treated specimens (Figure 16) displayed a more granular and porous fracture appearance with visible microvoids, compatible with EO-induced swelling and microcracking phenomena, in agreement with the observed reduction in elongation at break and the decrease in $T_{g}$.

The observed fracture morphology is therefore consistent with the mechanical and viscoelastic results obtained in this study. The HP treatment appears to promote a degree of superficial surface homogenization, as evidenced by smoother fracture facets, while inducing only moderate mechanical compromise reflected in reduced elastic modulus and impact resistance. In contrast, EO sterilization leads to more pronounced embrittlement and structural heterogeneity, characterized by granular fracture features and microvoid formation. These morphological characteristics correlate with the significant reduction in elongation at break, the decrease in $T_{g}$, and the overall softening of the viscoelastic response, confirming that each sterilization method affects the composite through distinct degradation mechanisms.

## 4. Discussion

### 4.1. Effects of Hydrogen Peroxide Sterilization (PC+CF-HP)

HP vapor sterilization exerted significant influence on the viscoelastic, thermal, and mechanical properties of the FDM-printed PC+CF specimens. Although the decrease in ultimate tensile strength was minor (from 53.0 ± 0.7 MPa to 52.2 ± 0.4 MPa), the elastic modulus dropped markedly (from 175.5 ± 5.0 MPa to 152.8 ± 4.4 MPa), suggesting a reduction in structural stiffness. This is likely due to interlaminar defects or void formation caused by oxidative degradation and thermal stress during the low-pressure sterilization cycle, consistent with previous observations in FDM polymers [29,30].

The Charpy impact resistance decreased significantly, further indicating the weakening of interlayer adhesion—an intrinsic vulnerability in FDM-printed parts. Interestingly, a moderate increase in elongation at break was observed, potentially caused by relaxation of residual stresses or localized plasticization due to HP exposure, which may soften the polymer surface without deep matrix penetration [31].

Despite the observed mechanical drawbacks, HP sterilization improved the thermal stability of the material when evaluated by thermomechanical analysis (TMA). The glass transition temperature ($T_{g,\mathrm{TMA}}$) increased from 136.6 °C in the unsterilized condition to 147.9 °C after HP treatment, accompanied by a reduction in the coefficient of thermal expansion (CTE), indicating enhanced dimensional stability under thermal loading. This behavior may be associated to molecular reorganization or minor cross-linking phenomena induced by peroxide exposure, as previously reported for peroxide-treated polycarbonates [32]. In contrast, dynamic mechanical analysis (DMA) revealed a slight shift of $T_{g}$ toward lower temperatures (from 118.38 °C to 114.73 °C), reflecting the higher sensitivity of oscillatory measurements to interfacial defects and microstructural heterogeneities inherent to FDM-printed composites. As TMA and DMA were conducted on a single specimen per condition, these interpretations remain indicative rather than conclusive.

DMA data further confirmed this behavior, showing a reduced initial storage modulus ($G^{\prime }$) of approximately 360 MPa compared with about 550 MPa for the unsterilized material and a leftward shift in tan(δ), implying a lower energy dissipation capacity. These findings suggest that HP sterilization promotes matrix densification but compromises elastic recovery, reinforcing the need to balance thermal integrity with mechanical reliability for clinical applications.

The results obtained show that hydrogen peroxide vapor sterilization causes a comprehensive modification in the behavior of FDM-printed PC+CF, simultaneously affecting its mechanical, thermal, and viscoelastic properties. The decrease in the elastic modulus and impact strength, together with the reduction in the storage modulus ($G^{\prime }$), reveals a generalized structural weakening, probably associated with interlaminar defects and microvoids generated by exposure to HP. Despite this, the increase in the glass transition temperature (*Tg*) and the decrease in the CTE indicate an improvement in thermal stability, possibly linked to molecular reorganization or surface crosslinking. The increase in elongation at break, although it could suggest greater ductility, seems to be related to localized plasticization or stress relaxation, rather than to a true improvement in the material’s toughness. Taken together, these findings reflect that HP alters the material’s characteristics in a complex and intertwined way, reinforcing the need to evaluate its overall behavior rather than just individual properties when considering its application in clinical settings.

### 4.2. Effects of Ethylene Oxide Sterilization (PC+CF-EO)

In contrast, ethylene oxide (EO) sterilization had a more deleterious effect on ductility and thermal performance. Although tensile strength was preserved, the elongation at break dropped significantly from 65.2 ± 3.2% to 51.9 ± 3.5%, denoting polymer embrittlement. This is likely due to EO absorption and plasticization, which disrupts intermolecular bonding and reduces mechanical cohesion—a mechanism supported by biomedical studies on EO-treated polymers [9,10,33].

The glass transition temperature determined by DMA decreased from 118.38 °C in the control sample to 114.32 °C after EO sterilization, and the CTE increased, indicating compromised thermal resistance. DMA confirmed these changes, with an initial storage modulus ($G^{\prime }$) of approximately 240 MPa and a tan(δ) peak shift to lower temperatures, suggesting softened viscoelastic response. These thermal alterations may be related to degradation processes such as plasticization or chain mobility changes reported for EO-treated polymers. The absence of significant FTIR spectral changes suggests that the observed mechanical degradation is primarily associated with physical effects such as molecular mobility changes or residual sterilant absorption rather than with detectable chemical modification of the polymer backbone, as also reported in similar studies analyzing EO-treated polymers using FTIR [28].

Furthermore, microscopic analysis revealed rough, porous fracture surfaces, potentially due to EO-induced swelling or microcracking. While a minor increase in surface hardness was recorded, this local change did not compensate for the observed reductions in ductility and thermal stability.

Overall, EO sterilization compromises both the mechanical resilience and thermomechanical integrity of PC+CF, making it a less favorable option for load-bearing or thermally sensitive medical applications.

Ethylene oxide (EO) sterilization negatively and comprehensively affected the properties of FDM-printed PC+CF, compromising its mechanical, thermal, and viscoelastic performance. Although tensile strength remained stable, the significant reduction in elongation at break indicates material embrittlement, likely due to EO absorption, which weakens intermolecular bonding and disrupts structural cohesion. This mechanical fragility was accompanied by thermal degradation, evidenced by a decrease in glass transition temperature (*Tg*) and an increase in the CTE, suggesting reduced heat resistance and greater dimensional instability. DMA results confirmed a weakened viscoelastic response, with a decrease in storage modulus ($G^{\prime }$) and a shift of the tan(δ) peak to lower temperatures, consistent with potential chain scission or partial depolymerization.

Morphologically, rough and porous fracture surfaces support the hypothesis of EO-induced swelling or microcracking. Although a slight increase in surface hardness was observed, this localized change does not compensate for the broader losses in ductility and thermal stability.

Overall, the findings indicate that EO sterilization compromises the global integrity of the material, making it a less favorable option for medical applications requiring structural and thermal reliability. However, given the single-specimen TMA/DMA measurements, these mechanisms should be interpreted as indicative rather than conclusive.

### 4.3. Implications for 3D-Printed Medical Device Design

These findings underscore the critical role of sterilization selection in the functional performance of FDM-printed components. HP vapor, despite some mechanical drawbacks, improves thermal stability and may be suitable for applications such as surgical guides, electronic housings, or components subjected to thermal fluctuations.

Conversely, EO treatment appears to degrade both thermal and mechanical properties, restricting its use unless additional stabilization or post-processing strategies are applied.

This study contributes novel empirical evidence for understanding sterilization-induced changes in PC+CF composites and supports more informed decision-making in material selection for additive manufacturing in clinical environments. Further studies using high-resolution techniques such as Fourier Transform Infrared Spectroscopy (FTIR) or SEM currently unavailable would help clarify the chemical degradation mechanisms and confirm surface-level changes. In addition, the inclusion of DMA provides a more nuanced view of viscoelastic alterations, reinforcing the value of dynamic testing protocols in assessing polymer suitability for sterilizable, high-performance medical applications. Medical devices are typically subjected to repeated sterilization throughout their service life. In the present work, material behavior was assessed after a single exposure in order to isolate the primary physicochemical mechanisms associated with each sterilization method.

This strategy enables separation of the intrinsic degradation mode linked to ethylene oxide diffusion from the oxidative effects induced by hydrogen peroxide plasma. For polymeric materials, the initial sterilization cycle commonly produces the most significant structural alteration, whereas subsequent cycles predominantly intensify pre-existing damage rather than introducing new governing mechanisms. Consequently, the single-cycle response can be considered representative of the dominant degradation pathway controlling long-term performance. Future studies will address cyclic sterilization to determine damage accumulation kinetics and to define clinical service limits for additively manufactured PC–CF components.

## 5. Conclusions

This study evaluated the effects of two low-temperature sterilization methods—ethylene oxide (EO) and hydrogen peroxide vapor (HP)—on the mechanical, thermal, and viscoelastic behavior of fused deposition modeling (FDM)-printed carbon fiber-reinforced polycarbonate (PC-CF).

HP sterilization produced a moderate reduction in mechanical performance, particularly in elastic modulus and impact resistance, suggesting partial alteration of the interlaminar architecture inherent to additively manufactured structures. However, thermomechanical analysis (TMA) revealed improved thermal stability after HP treatment, characterized by an increase in glass transition temperature ($T_{g}$) and a reduction in the coefficient of thermal expansion (CTE), indicating enhanced dimensional stability under thermal loading conditions.

In contrast, EO sterilization caused more pronounced deterioration in ductility and thermal resistance. The significant reduction in elongation at break, accompanied by a decrease in $T_{g}$ and an increase in CTE, indicates embrittlement and reduced thermomechanical stability, likely associated with EO-induced plasticization and increased chain mobility within the polymer matrix.

Dynamic mechanical analysis (DMA) confirmed that both sterilization processes modified the viscoelastic response of PC-CF, as evidenced by reductions in storage modulus ($G^{\prime }$) and shifts in the tan(δ) curves. While HP-treated specimens retained comparatively better thermal resilience, EO-treated samples exhibited more pronounced viscoelastic softening.

Overall, the observed sterilization-induced changes are strongly influenced by the 80% infill additively manufactured architecture, where interlayer bonding and internal porosity significantly contribute to mechanical and thermomechanical behavior. The integration of macro-mechanical testing (N=5) with thermomechanical, viscoelastic, and microstructural analyses provides a multi-scale framework for assessing degradation mechanisms in sterilized 3D-printed composites.

These findings highlight the importance of sterilization-aware design in polymer-based medical devices fabricated via additive manufacturing and provide foundational data to support material selection, regulatory evaluation, and performance qualification. Future work should incorporate advanced surface and chemical characterization techniques, such as Fourier transform infrared spectroscopy (FTIR) and scanning electron microscopy (SEM), to further elucidate molecular degradation mechanisms and explore strategies to enhance sterilization resistance.

Study limitations: It is important to acknowledge that the intrinsic variability associated with the FDM process would ideally require larger sample sizes for thermal characterization. In this study, the thermomechanical analysis (TMA) and DMA results were obtained with a limited number of specimens and should therefore be interpreted as indicative trends in material response rather than as statistically representative values. While the consistency observed with the mechanical data supports the proposed degradation mechanisms, these findings remain exploratory in nature. Future investigations with expanded experimental resources should incorporate larger sample sets to enable rigorous statistical analysis, including significance testing (e.g., ANOVA), thereby strengthening the quantitative robustness of the conclusions.

## Figures and Tables

**Table 1:** Sample nomenclature for PC-CF under different sterilization conditions.

Material	Unsterilized Specimens	Specimens Sterilized with Ethylene Oxide	Specimens Sterilized with Hydrogen Peroxide
Carbon Fiber–Reinforced Polycarbonate	PC+CF	PC+CF-EO	PC+CF-HP

**Table 2:** Mechanical properties of FDM-printed carbon fiber–reinforced polycarbonate before and after EO and HP sterilization.

Average Sample Values					
Material	Ultimate Tensile Strength	Elastic Modulus	Elongation at Break	Charpy Impact Strength	Shore Hardness (D)
	(MPa)	(MPa)	(%)	(kJ/m^2^)	(HD)
PC+CF	53.0 ± 0.7 ^a^	175.5 ± 5.0 ^a^	65.2 ± 3.2 ^a^	29.3 ± 1.3 ^a^	78.1 ± 0.1 ^b^
PC+CF-HP	52.2 ± 0.4 ^a^	152.8 ± 4.4 ^a^	69.9 ± 2.9 ^a^	22.7 ± 1.3 ^b^	79.1 ± 0.1 ^a^
PC+CF-EO	52.2 ± 0.4 ^a^	199.9 ± 20.9 ^a^	51.9 ± 3.5 ^b^	22.7 ± 1.6 ^b^	79.2 ± 0.1 ^a^

Note. a,b Different letters within the same property indicate statistically significant differences between sterilization processes (p<0.05), according to Tukey’s pairwise comparison.

**Table 3:** Glass transition temperature and linear thermal expansion coefficients (CTE1 and CTE2) for each group.

TMA Results			
Material	Glass Transition Temperature ($T_{g}$)	Linear CTE 1 Before $T_{g}$	Linear CTE 2 After $T_{g}$
	(°C)	(µm/(m·°C))	(µm/(m·°C))
PC+CF	136.57	127.9 @ 90 °C	306.0 µm/(m·°C) @ 200 °C
PC+CF-EO	126.88	131.4 @ 80 °C	330.9 µm/(m·°C) @ 200 °C
PC+CF-HP	147.86	105.5 @ 70 °C	277.4 µm/(m·°C) @ 200 °C

Note. Values represent individual measurements (*N* = 1) indicating behavioral trends.

## Data Availability

The original contributions presented in this study are included in the article. Further inquiries can be directed to the corresponding author.
